# Acquisition of New Migratory Properties by Highly Differentiated CD4+CD28^null^ T Lymphocytes in Rheumatoid Arthritis Disease

**DOI:** 10.3390/jpm11070594

**Published:** 2021-06-24

**Authors:** Beatriz Rioseras, Marco Antonio Moro-García, Alejandra García-Torre, Eva Bueno-García, Rocio López-Martínez, Maria Iglesias-Escudero, Roberto Diaz-Peña, Patricia Castro-Santos, Miguel Arias-Guillén, Rebeca Alonso-Arias

**Affiliations:** 1 Immunology Department, Medicine Laboratory, Hospital Universitario Central de Asturias, 33011 Oviedo, Spain; brioseras@gmail.com (B.R.); san.torre15@gmail.com (A.G.-T.); evabugar@gmail.com (E.B.-G.); rociolopez-@hotmail.com (R.L.-M.); 2 Health Research Institute of the Principality of Asturias—ISPA, 33011 Oviedo, Spain; marcomorog@hotmail.com; 3 Medicine Laboratory, Hospital Universitario Central de Asturias, 33011 Oviedo, Spain; 4 Immunology Department, Hospital Universitario German Trias i Pujol, 08916 Badalona, Spain; mar.iglesias.86@gmail.com; 5 Faculty of Health Sciences, Universidad Autónoma de Chile, Talca 3460000, Chile; roberto.diaz@uautonoma.cl; 6 Inmunologia, Centro de Investigaciones Biomédicas (CINBIO), Universidad de Vigo, 36310 Vigo, Spain; patricia.castro@uautonoma.cl; 7 Servicio de Neumología, Hospital Universitario Central Asturias, 33011 Oviedo, Spain; miguelariasguillen@gmail.com; 8 CIBER—Enfermedades Respiratorias, Instituto de Salud Carlos III, 28029 Madrid, Spain

**Keywords:** CD4+CD28^null^ T lymphocytes, rheumatoid arthritis, IL-15, migration

## Abstract

Expanded CD4+CD28^null^ T lymphocytes are found in the tissues and peripheral blood of patients with many autoimmune diseases, such as rheumatoid arthritis (RA). These highly differentiated cells present potent inflammatory activity and capability to induce tissue destruction, which has been suggested to predispose to the development of more aggressive disease. In fact, preferential migration to inflammatory sites has been proposed to be a contributing factor in the progression of autoimmune and cardiovascular diseases frequently found in these patients. The functional activity of CD4+CD28^null^ T lymphocytes is largely dependent on interleukin 15 (IL-15), and this cytokine may also act as a selective attractor of these cells to local inflammatory infiltrates in damaged tissues. We have analysed, in RA patients, the migratory properties and transcriptional motility profile of CD4+CD28^null^ T lymphocytes compared to their counterparts CD28+ T lymphocytes and the enhancing role of IL-15. Identification of the pathways involved in this process will allow us to design strategies directed to block effector functions that CD4+CD28^null^ T lymphocytes have in the target tissue, which may represent therapeutic approaches in this immune disorder.

## 1. Introduction

After their maturation in the thymus, the naïve CD4+ T lymphocytes are activated through antigen recognition. This recognition consists of the binding of TCR and CD4 receptors with MHC class II, which carries the specific antigen on the surface of antigen presenting cells (APCs). However, this interaction is not enough to trigger an immune reaction since a co-stimulatory signal is necessary. There are different co-stimulatory molecules that allow this process, namely, CD28 receptors in T lymphocytes being the most important [1]. These co-stimulatory signals can also regulate the cytoskeleton rearrangement and adhesion of T lymphocytes. Therefore, it is known that apart from being critical for the activation, proliferation, and survival of CD4+ T lymphocytes, CD28 is also involved in the migration process of these cells [2].

The lack of this CD28 molecule during activation results in partial activation or even an anergic state of T lymphocytes. However, this is not the case with CD4+CD28^null^ T lymphocytes that, instead of being anergic, have enhanced effector functions increasing their inflammatory features, such as cytokine production (IFNγ an TNF), accumulation of molecules involved in cellular cytotoxicity (perforin and granzyme), expression of NK cell-related receptors (NKRs), loss of their sensitivity to apoptosis induction or their resistance to the suppressive actions of regulatory T (Treg) cells [3].

It is known that these CD4+CD28^null^ T lymphocytes are expanded in some situations not only as an immunosenescence process [4] but also under other clinical conditions involving chronic activation of the immune system, such as viral infections or autoimmune diseases [5]. In rheumatoid arthritis (RA), it has been specifically shown that CD4+CD28^null^ T lymphocytes are significantly increased in the blood and synovial fluid of these patients, and it has been suggested that these cells have an active role in the maintenance of inflammation [6,7]. In some of these situations where CD4+CD28^null^ T lymphocytes are expanded, it has been demonstrated that interleukin 15 (IL-15) has a role in their proliferation and enhances the effector response of CD4+CD28^null^ T lymphocytes against their specific chronic antigens [8,9]. In fact, elevated serum and synovial fluid levels of IL-15 have been detected in RA patients, suggesting an important role of this cytokine in disease immunopathology and a correlation with clinical activity [10,11]. As a result, IL-15 has been recently proposed as a biomarker candidate for prediction of the development of RA, especially in the early phase of disease [12].

Although the characteristics of CD4+CD28^null^ T lymphocytes have been widely studied in these pathologic situations, little is known about the migration patterns that they use to reach the target tissue perpetuating the damage. The migration of mature T lymphocytes consists of their recirculation through the blood to lymph nodes in order to facilitate the encounter with their specific antigen. This situation happens in physiologic conditions, but when a tissue is infected, T lymphocytes can migrate and go into the target tissue. Different adhesion molecules, such as integrins (LFA1, VLA-4), chemokine receptors (CX3CR1, CCR5) and hyaluronic acid receptors (CD44), as well as Rho-GTPase proteins that control the cytoskeleton dynamics of these cells (RhoA, Rac1/2, Cdc42), are essential for this process [13,14,15]. Besides these proteins, there are a high number of genes that are regulating complex pathways related to the migration process, such as focal adhesion, leucocyte extravasation and actin cytoskeleton regulation. With respect to CD4+CD28^null^ T lymphocytes, as we have advanced above, they have been identified not just in the circulation of patients with inflammatory diseases but also in target tissues, such as the joints of RA patients [6]. Some studies suggest that expression of chemokine receptors and adhesion molecules by CD4+CD28^null^ T lymphocytes could have an important role in guiding their migration and ability to infiltrate tissues, which allow these cells to cause local inflammation and tissue damage [3].

The goal of this work is to study the adhesion and migration abilities of CD4+CD28^null^ T lymphocytes in RA patients, as well as the effect that IL-15 could have in these processes, in order to find possible candidates for therapeutic targets.

## 2. Materials and Methods

### 2.1. Donors

Peripheral blood samples were obtained from 65 donors with a diagnosis of RA according to the American College of Rheumatology (ACR) and the European League Against Rheumatism (EULAR) classification criteria in the Hospital Universitario Central de Asturias (Oviedo, Spain). All these RA patients selected for the study were in the age range of 40–65 and had positive anti-cyclic citrullinated peptide (anti-CCP) and/or rheumatoid factor (RF) tests. The threshold for considering these values positive were ≥3 U/mL for anti-CCP and >14 Ku/L for RF. Experiments were performed with blood samples from those individuals whose percentage of CD4+CD28^null^ T lymphocytes was 5% or higher of total CD4+ T lymphocytes. The different experiments focused on analysing the factors associated with the migratory ability of CD4+CD28^null^ T lymphocytes were performed with 41 of these donors that fulfilled this inclusion criterium. The study was approved by the Hospital Central de Asturias (Oviedo, Spain) Ethics Committee.

The ethics committee approved the exemption of informed consent in this study since only excesses of peripheral blood (intended to be destroyed) extracted for clinical purposes were used and never for the purpose of being used in research. The samples were anonymized from the first moment after clinical studies necessary for the clinical follow-up of RA disease were performed, and there was no record in which the origin of the samples could be determined. 

### 2.2. Quantification of CD4+CD28^null^ T Lymphocytes

The percentage of CD4+CD28^null^ T lymphocytes was determined by staining with anti-CD3 (FITC), anti-CD28 (PE) (eBioscience, San Diego, CA, USA), anti-CD8 (PerCP), and anti-CD4 (APC) (Immunostep, Salamanca, Spain) monoclonal antibodies. A total of 100 µL of whole blood from the donors were stained with the combination of labelled monoclonal antibodies for 30 min at room temperature. Samples were red blood lysed for 10 min at room temperature with FACS Lysing Solution (BD Biosciences, San José, CA, USA), washed in PBS, and analysed with BD Accuri C6 Software in the Accuri C6 (BD Biosciences, San José, CA, USA).

### 2.3. Cell Isolation and Culture Conditions

Peripheral blood mononuclear cells (PBMCs) were isolated from peripheral blood that had been anticoagulated with EDTA by centrifugation on Ficoll–Hypaque gradients (Lymphoprep, Nycomed, Oslo, Norway).

CD4+ T lymphocytes were enriched by the incubation of the peripheral blood anticoagulated with EDTA with the RosetteSep™ Human CD4+ T Cell Enrichment Cocktail (STEMCELL, Vancouver, Britids Columbia, Canada) for 20 min at room temperature. After this incubation, CD4+ T lymphocytes were obtained by centrifugation on Ficoll-Hypaque gradients (Lymphoprep; Nycomed).

The CD4+ purified cells were separated into CD4CD28+ and CD4+CD28^null^ using the CD28 MicroBead Kit and the separation columns MACS MS (MiltenyiBiotec, Bergisch Gladbach, Germany). 

According to manufacturer instructions, starting with fresh human whole blood, the CD4+ T cell content of the enriched fraction is typically 94 ± 5% (mean ± SD). Specifically, in our experiments, the purity of both CD4, CD28+, and CD28null isolated cells was evaluated by flow cytometry, and it was never lower than 95%.

Cultures were performed in RPMI 1640 medium containing 2 × 10^−3^ M l-glutamine and Hepes (BioWhitaker, Verviers, Belgium) and supplemented with 10% FCS (ICN Flow; Costa Mesa, CA, USA) and antibiotics. Cells at a concentration of 2 × 10^6^ cells/mL were incubated at 37 °C and 5% carbon dioxide.

### 2.4. Expression of Chemokine Receptors and Adhesion Molecules

Basal expression of the different molecules was determined in peripheral blood obtained from seven RA patients, as described above. The effect of IL-15 on the expression of these molecules was studied in PBMCs obtained from 10 RA patients and cultured in medium alone or in the presence of IL-15 (50 ng/mL) (Peprotech INC, Rockyhill, NJ, USA) for 18 h. Overnight treatment with IL-15 has been seen to be the appropriate frame time for protein expression detection in previous studies [8,16]. The surface stain in both cases was made with anti-CD4 (APC) or anti-CD4 (PECy7), anti CD45RA (APCFire), anti-CD8 (PB), anti-CD28 (BV), anti-CD44 (FITC), anti-CX3CR1 (PE), anti-CCR5 (PECy7), and anti-CD11a (FITC) (Biolegend, San Diego, CA, USA), as well as anti-CD3 (PerCP) and anti-CD49d (APC) (BD Bioscience). Cells from whole blood were stained as described above. PBMCs were stained for 30 min at 4 °C. Then, cells were washed and resuspended in PBS until they were acquired in a Navios flow cytometer and analysed with Kaluza software (Beckman Coulter Life Science, Brea, CA, USA). The cytometer compensation was carried out using the VersaComp Antibody Capture Bead Kit (Beckman Coulter). The marker settings for determining the negative/positive cell populations were established using the FMO strategy.

### 2.5. Cell Migration Assays

Cell migration assays were performed with CD4+ T lymphocytes isolated from eight patients using a transwell system with a pore size of 3 µm (Costar, Kennebunk, ME, USA). Briefly, between 2 × 10^5^ and 5 × 10^5^ CD4+ T lymphocytes in 100 µL of medium were placed in the upper chamber of a transwell system suspended over a larger well containing 600 µL of culture medium or in the presence of IL-15 (50 ng/mL). Cells were allowed to migrate through the pores to the other side of the membrane. After 18 h, migratory cells (MCs) and non-migratory cells (NMCs) were collected. Then, they were surface stained with anti-CD45RA (FITC) (Biolegend), anti-CD28 (PE), and anti-CD4 (PerCP) (BD Bioscience) monoclonal antibodies. Flow cytometric analysis was made using the Accuri C6 (BD Biosciences). For the cell count, 20 µL of the sample was acquired at medium speed. We have tested different concentration of IL-15 (from 0.5 to 100 ng/mL) to perform these transwell assays and we have observed a significant increment in the cell migration capacity with all the IL-15 concentrations tested comparing to unstimulated conditions. The maximum peak of migration was already reached for both T CD4+ lymphocyte subsets studied with 50ng/mL (data not shown). Based on this, we decided to use this IL-15 concentration for the rest of experiments.

### 2.6. Migration RT-PCR Array

Isolated CD4+CD28+ and CD4+CD28^null^ T lymphocytes from eight patients were pooled and cultured in medium alone or in the presence of IL-15 stimulation (50 ng/mL) for 4 h. We have chosen 4 h as the time to perform the gene expression experiments based on the previous knowledge about PBMCs gene expression after its stimulation in cell culture and on data from our own experience [16]. mRNA from these cells was extracted using the RNeasy Mini Kit (Qiagen, Düsseldorf, Germany) according to the manufacturer’s instructions, including the optional on-column DNase digestion step with RNAse-Free DNase set (Qiagen). Reverse transcription of mRNA isolated from each sample was carried out in a 20 μL final volume with the cDNA Synthesis Using the RT2 First Strand Kit (Qiagen), as indicated in the instructions provided by the manufacturer. cDNA was stored at −80 °C until required for RT-PCR array. An RT-PCR array of 96 wells was performed for each cDNA sample following manufacturer’s instructions (PAHS-128Z RT2 ProfilerTM PCR Array Human Cell Motility, Qiagen) and analysed using a 7500 Real Time PCR System (Applied Biosystem, Foster City, CA, USA).

RT-PCR array analysis was conducted at Qiagen’s GeneGlobe Data Analysis Center using a software tool available on the website. 

All the samples passed the three control tests performed before the analysis: PCR array reproducibility, reverse transcription efficiency, and genomic DNA contamination. Two constitutively expressed genes, glyceraldehide-3-phosphate dehydrogenase and beta-2-microglobulin, were used for data normalization.

We compared two different conditions: CD4+CD28^null^ (test sample) vs. CD4+CD28+ (control sample) and CD4+CD28^null^ IL-15 stimulation (test sample) vs. CD4+CD28^null^ basal conditions (control sample). Fold change 2^(ΔΔCT) that is the normalized gene expression (2^(−ΔCT)) in the test sample dividing the normalized gene expression (2^(−ΔCT)) in the control sample was calculated for each of the compared conditions.

In order to represent fold change results in a biologically meaningful way, fold regulation was calculated. When fold change values are greater than one, it indicates an upregulation and fold regulation is equal to the fold change in these cases. However, when fold change values are less than one, it indicates a downregulation, and fold regulation is calculated as the negative inverse of the fold change. Genes were considered significantly upregulated when the fold regulation was greater than 2 and downregulated when the fold regulation was lower than −2.

Log_2_ of the fold change was calculated for the heat maps representation and pathway analysis in order to normalize and get a better visualization of the data. The pathway-based data integration and visualization was performed using the pathview R package (1.30.1 version) from the bioconductor bioinformatics repository [17].

### 2.7. Activity Quantification of Rho GTPases Involved in Cellular Migration

Isolated CD4+CD28+ and CD4+CD28null T lymphocytes from eight patients were pooled and cultured in serum-free conditions for 1 h. After this, lymphocytes were cultured in completed medium alone or in the presence of IL-15 stimulation (50 ng/mL) for 5 min. It was well documented that the GTPases activation occurs in a very short period of time (a few minutes), and as it is said in the manufacturer instructions of G-LISA activation assays (Cytoskeleton, Inc., Denver, CO, USA), Rho proteins are generally activated very rapidly and transiently (30 s to 30 min). Following this, we decided to incubate under IL-15 stimulation for 5 min. After the incubation, cold PBS was added to the cells in order to stop the stimulation. Protein extracts were obtained from each condition and both basal levels of RhoA and activated forms of RhoA, Rac1, and Cdc42 were measured following the manufacturer instructions using G-LISA activation assays (Cytoskeleton, Inc., Denver, CO, USA). Calpeptine was used as the positive control for RhoA activation.

### 2.8. Statistical Analysis

Comparisons between groups were performed with the nonparametric Wilcoxon signed-rank test when data was not normally distributed or with Student’s t test for paired data when data was normally distributed. To compare the migratory abilities between different conditions, data were logarithmically transformed and a post hoc multiple comparisons with Bonferroni adjustment was performed. Analyses were performed using the PASW Statistics 17.0 statistical software package (IBM SPSS, Armonk, NY, USA), and *p*-values of 0.05 or less were considered significant.

## 3. Results

### 3.1. Adhesion Molecules and Chemokine Receptor Expression in CD4+CD28^null^ and D4+CD28+ T Memory Cells at Basal Conditions and after IL-15 Stimulation

We evaluated the expression levels of different proteins involved in the migration of CD4+ T lymphocytes from RA patients. The molecules analysed were CD11a and CD49d, which are chains that form part of LFA1 and VLA-4 integrins, respectively, CX3CR1 and CCR5 chemokine receptors, and the hyaluronic acid receptor CD44. The expression of these molecules in the two different subsets of CD4+ memory T lymphocytes, CD4+CD28+CD45RA- T lymphocytes and CD4+CD28^null^ T lymphocytes, was compared.

**Figure 1:**
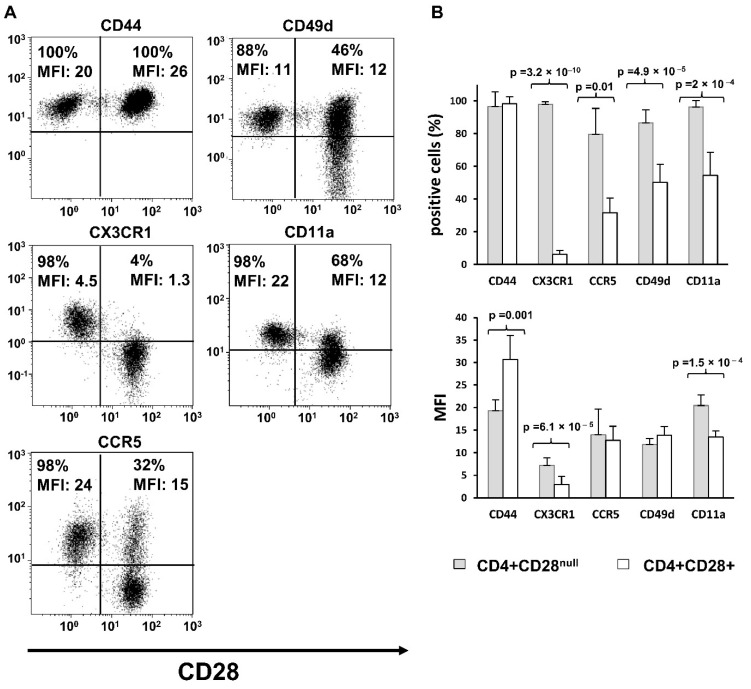
Basal expression of adhesion molecules and chemokine receptors. Results are representative for peripheral blood of seven RA patients. Expression levels of CD44, CX3CR1, CCR5, CD49d, and CD11a were analysed in both CD4+CD28+ and CD4+CD28^null^ T cells by flow cytometry. (**A**) Dot-plots show a representative experiment; CD28 stain is represented on the *X* axis. The percentage of positive cells and MFI for each molecule is displayed in the figure. (**B**) Histograms represent the percentage of positive cells and MFI (mean ± SD) in CD28+ and CD28^null^ cells. Paired T tests were used to compare differences; significant differences are indicated on the panel.

The percentage of cells with basal expression of CX3CR1, CCR5, CD49d, and CD11a were significantly higher in CD4+CD28^null^ T lymphocytes compared to that in CD4+CD28+ T lymphocytes (paired T test, *p* = 3.2 × 10^−10^, *p* = 0.01, *p* = 4.9 × 10^−5^, and *p* = 2 × 10^−4^, respectively). When mean fluorescence intensity (MFI) of the positive populations was compared, a similar significant increment in expression for CX3CR1 and CD11a in CD4+CD28^null^ with respect to CD4+CD28+ T lymphocytes was found (paired T test, *p* = 6.1 × 10^−5^, *p* = 1.5 × 10^−4^, respectively). The CD4+CD28^null^ T lymphocyte population, besides having more cells expressing these two molecules at basal conditions, also has an increased expression level of these molecules per cell. No differences were detected in the positive population percentage of CD44 between the two CD4+ T lymphocyte subsets due to almost 100% of the cells expressing this molecule at basal conditions in both CD4+ T lymphocyte subsets. However, the CD44 MFI was significantly higher in CD4+CD28+ T lymphocytes than in CD4+CD28^null^ T lymphocytes (paired T test, *p* = 0.001) (Figure 1).

**Figure 2:**
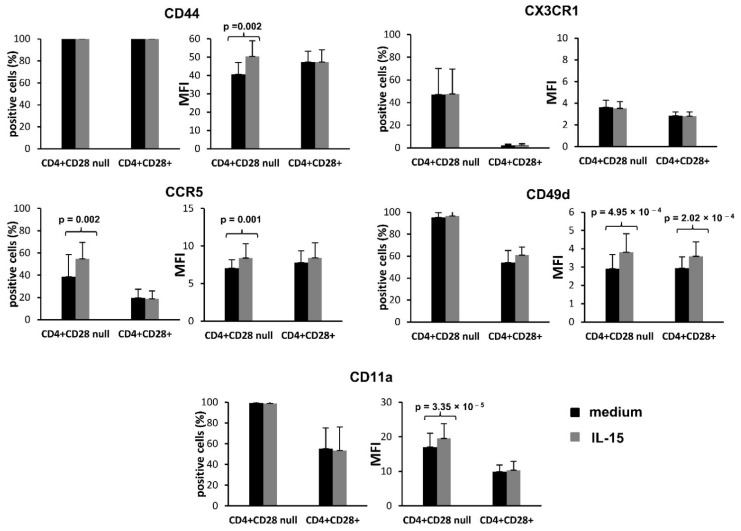
Effect of IL-15 on the expression of migration-related proteins. The results are representative of 10 RA patients. Peripheral blood mononuclear cells were cultured for 18 h in the presence or absence of IL-15 (50 ng/mL). Expression levels of CX3CR1, CD44, CCR5, CD11a, and CD49d were analysed in CD4+CD28+ and CD4+CD28^null^ T cells by flow cytometry. Histograms represent the percentage and MFI of positive cells (mean ± SD) in CD28+ and CD28^null^ cells cultured in medium (black bars) and in the presence of IL-15 (grey bars). Paired T tests were used to compare differences; significant differences are indicated on the panel.

To further evaluate the different behaviour of these two CD4+ memory T lymphocyte subsets, the expression of these molecules was also measured in PBMCs incubated in culture medium in the absence and presence of IL-15 (50 ng/mL) (Figure 2). 

A significant increase was detected in the expression level of CD44, CCR5, CD49d, and CD11a in the CD4+CD28^null^ T lymphocytes when they were stimulated with IL-15 in comparison with basal culture conditions (paired T test, CD44: *p* = 0.002, MFI; CCR5: *p* = 0.002, positive cell percentage and *p* = 0.001, MFI; CD49d: *p* = 4.9 × 10^−4^, MFI; CD11a: *p* = 3.35 × 10^−5^, MFI). This effect of IL-15 on CD49d expression was also observed in CD4+CD28+ T lymphocytes (paired T test, *p* = 2.02 × 10^−4^, MFI), but no significant differences were shown on CCR5, CD11a, or CD44 expression in this T lymphocyte subset. Notice that, as it happened with the basal expression of CD44, in cases where the positive population for the studied molecule is already very high (close to 100%) in cells cultured without stimulation (CD44, CD49d, and CD11a), the increment in expression level was not significant when the expression is reflected as the percentage of positive cells, but it is clearly apparent when the expression level is measured as the MFI of the positive population. Ultimately, no significant changes were detected in the CX3CR1 expression between basal conditions and IL-15 stimulation in neither the CD4+CD28^null^ nor the CD4+CD28+ T lymphocyte subset (Figure 2).

### 3.2. CD4+CD28^null^ and CD4+CD28+ Migratory Ability in Chemotaxis Assays

**Figure 3:**
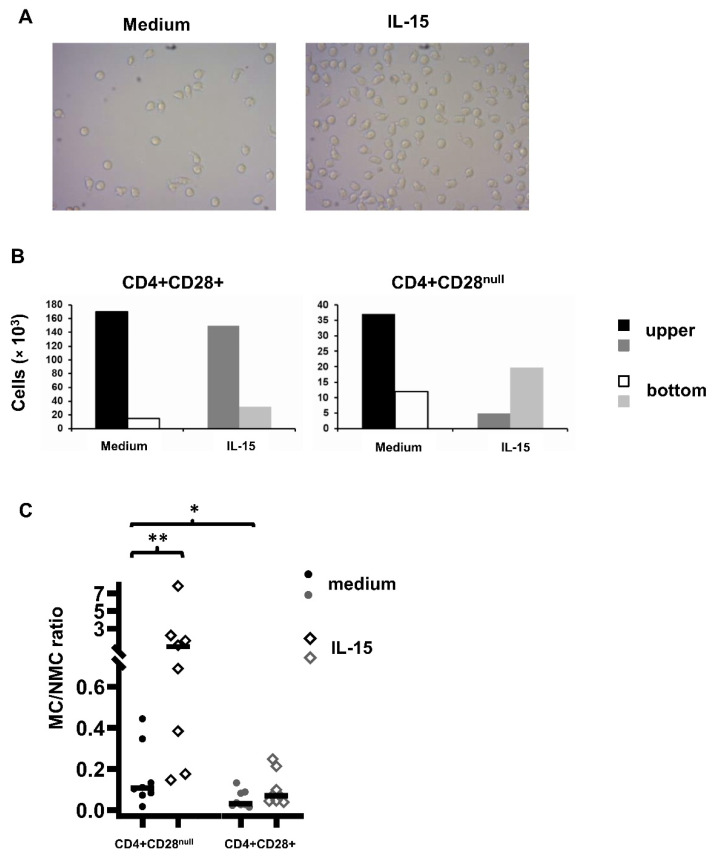
Transwell migration assay of CD4+ T lymphocytes in culture medium alone and in the presence of IL-15 (50 ng/mL) in 18 h culture. (**A**) CD4+ T lymphocytes in the lower compartment of a transwell system in culture medium alone and in culture medium plus IL-15. Photographs were taken with an inverted microscope at 100X after examining 10 different areas of each lower compartment in different experiments with similar results. (**B**) Representative experiment. Histograms represent the number of non-migrating cells (NMCs) and the number of migrating cells (MCs) in the presence or absence of IL-15. The NMCs are those that, after 18 h of incubation, have remained in the upper compartment of the transwell system, and the MCs are those that, after that time, have crossed the membrane and passed to the lower compartment of the transwell system. (**C**) Ratio between MCs and NMCs in the two conditions and at 18 h of culture. CD4+ T lymphocytes isolated from peripheral blood samples were deposited in the upper compartment, and in the lower compartments, culture medium alone or culture medium plus IL-15 was added. After the incubation time, the cells were labelled with anti-CD45RA (FITC), anti-CD28 (PE), and anti-CD4 (PerCP). The fraction of CD4+CD28+ and CD4+CD28^null^ memory T lymphocytes was determined with Kaluza software; * shows significant differences between CD4+CD28+ and CD4+CD28^null^l memory T lymphocytes (*p* = 0.044); ** shows significant differences between CD4+CD28^null^ T lymphocytes cultured in medium and in the presence of IL-15 (*p* = 0.003).

To compare the migratory ability between CD4+CD28+ and CD4+CD28^null^ T lymphocytes cultured in medium and in response to IL-15, chemotaxis assays were carried out in transwell systems with a pore size of 3 µm. In the upper compartment, CD4+ T lymphocytes isolated from the peripheral blood of RA patients were added, whereas the lower compartment was filled with culture medium only or with IL-15 (50 ng/mL). Cultures were incubated at 37 °C and 5% CO_2_ for 18 h. In all the experiments, the number of CD4+ T lymphocytes in the bottom compartment was higher when IL-15 was present in the cultures. Figure 3 shows two representative images of the bottom compartments of the transwell system with medium alone and with IL-15 added (Figure 3A). They were taken after examining 10 different areas of each compartment in different experiments with similar results.

Cells from both compartments were collected and surface labelling was carried out. Once labelled, the cells were counted with the BD AccuriTM C6 cytometer (BioSciences). Absolute counts of CD4+CD28+ and CD4+CD28^null^ memory T lymphocytes in migrated and non-migrated fractions were quantified. Results from a representative patient are shown in Figure 3B. The migratory capacity of CD4+CD28^null^ T lymphocytes was significantly higher than that of CD4+CD28+ memory T lymphocytes. The median value of proportion of MCs vs. NMCs in the medium was 0.1 (IQR: 0.2) in CD4+CD28^null^ T lymphocytes vs. 0.03 (IQR: 0.06) in CD4+CD28+ memory T lymphocytes. The MC/NMC ratio showed a high variability between experiments, and the data was not distributed normally. To compare the migratory ability between different conditions, data were logarithmically transformed and post hoc multiple comparisons with Bonferroni adjustment were performed. Significant differences were found when the MC/NMC ratio was compared between CD4+CD28^null^ and CD4+CD28+ memory T lymphocytes (*p* = 0.044), as well as between CD4+CD28^null^ T lymphocytes cultured in medium or in the presence of IL-15. This cytokine showed an enhancing effect on CD4+CD28^null^ T lymphocyte migration (median rate MC/NMC: 0.9, IQR: 1.8) with respect to cells cultured in medium (*p* = 0.003), but the differences did not reach statistical significance in CD4+CD28+ memory T lymphocytes (median rate MC/NMC: 0.1, IQR: 0.14).

Pre-coating the transwell membrane with fibronectin, as well as direct treatment of CD4+ T lymphocytes with IL-15 during the culture instead of adding the cytokine in the bottom compartment, did not induce differences in migration (data not shown).

### 3.3. Analysis of Gene Expression Related to Cell Motility in CD4+CD28^null^ and CD4+CD28+ T Lymphocytes

**Figure 4:**
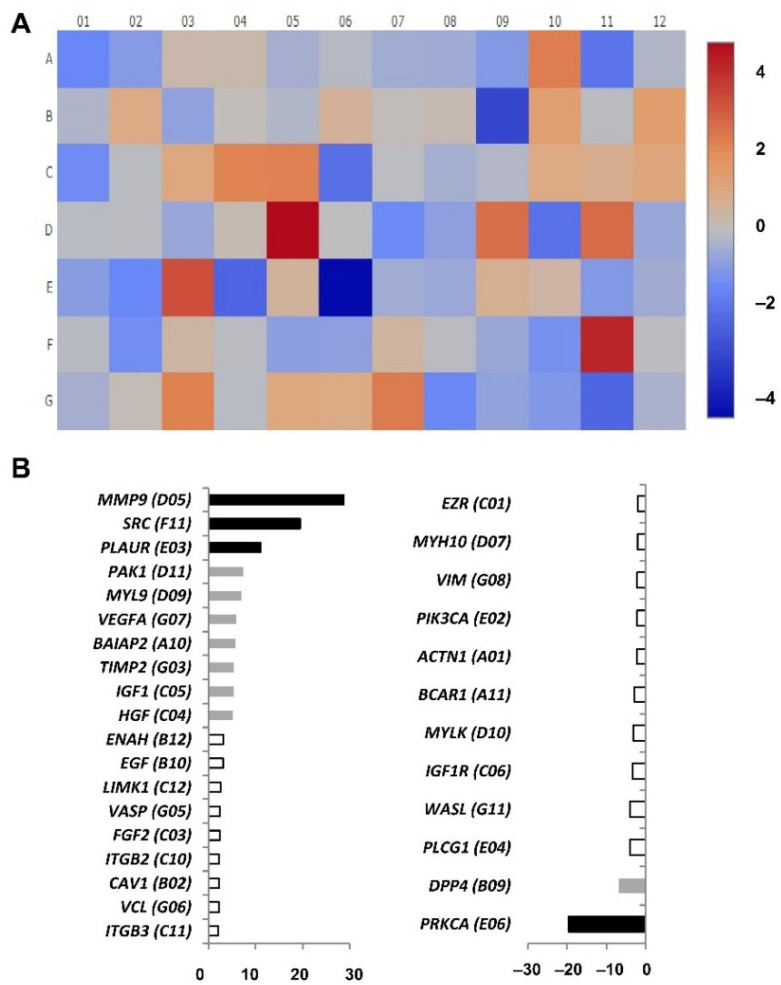
Cell motility array results comparing CD4+CD28^null^ T lymphocytes (test sample) with CD4+CD28+ T lymphocytes (control sample) testing the expression of 84 different genes. (**A**) Heatmap performed by calculating the log_2_ of the fold change for each gene. The figure represents the level of expression of each gene with a colour scale from dark red (more expression in CD4+CD28^null^) to dark blue (less expression in CD4+CD28^null^). (**B**) Representation of the genes with significant expression differences; upregulated genes on the left and downregulated genes on the right. Black bars represent the fold regulation of genes with an expression difference higher than 10, grey bars higher than 5 and white bars higher than 2. The wells corresponding to each gene are in brackets.

To analyse gene expression, cDNA pools were prepared from CD4+CD28^null^ and CD4+CD28+ T lymphocytes isolated independently from eight RA patients. Before RNA extraction, cells were both cultured in medium and stimulated for 4 h with IL-15. RT-PCR arrays were performed for the gene expression analysis of 84 different genes related to human cell motility in the four pools (Table S1). In order to determine the genes differentially expressed between CD4+CD28^null^ and CD4+D28+ T lymphocytes, fold regulation was calculated, taking CD4+CD28^null^ T lymphocytes as the test sample and CD4+CD28+ T lymphocytes as the control sample (Figure 4).

Nineteen of the tested genes showed significantly higher levels of expression in CD4+CD28^null^ T lymphocytes with respect to CD4+CD28+ T lymphocytes (fold regulation > 2) (Figure 4A). Among them, there were important genes involved in human cell migration, such as *MMP9* (matrix metallopeptidase 9), *SRC* (proto-oncogene, non-receptor tyrosine kinase), and *PLAUR* (urokinase-type plasminogen activator receptor), that were expressed in CD4+CD28^null^ T lymphocytes more than 10 times with respect to CD4+CD28+ T lymphocytes (Figure 4B). Other genes were expressed in CD4+CD28^null^ T lymphocytes more than five times, including *PAK1* (p21 activated kinase 1, which is the target for the small GTP binding proteins Cdc42 and Rac), *MYL9/MLC* (myosin light chain 9), *VEGFA* (vascular endothelial growth factor A), *BAIAP2* (BAR/IMD domain containing adaptor protein 2, which is associated with a downstream effector of Rho small G proteins), *TIMP2* (TIMP metallopeptidase inhibitor 2), *IGF1* (insulin-like growth factor 1), and *HGF* (hepatocyte growth factor) (Figure 4B). The rest of the upregulated genes in CD4+CD28^null^ T lymphocytes had an increase of expression from two to five times with respect to CD4+CD28+ T lymphocytes, including *ENAH/MENA* (ENAH actin regulator, which is an enabled/vasodilator-stimulated phosphoprotein involved in actin-based motility), *EGF* (epidermal growth factor), *LIMK1* (LIM domain kinase 1, which is involved in regulation of actin polymerization), *VASP* (vasodilator stimulated phosphoprotein), *FGF2* (fibroblast growth factor 2), *ITGB2* and *ITGB3* (integrin subunits beta 2 and 3, respectively), *CAV1* (caveolin 1), and *VCL* (vinculin) (Figure 4B).

On the other hand, 12 significantly downregulated genes in CD4+CD28^null^ T lymphocytes compared with respect to CD4+CD28+ T lymphocytes (fold regulation < −2) were detected. Notice that the difference of the expression in these genes is quite lower than in the upregulated genes (Figure 4A). Just one of these genes, *PPKCA/PKCA* (protein kinase C alpha), which is known to be involved in the regulation of many different cellular processes, showed an expression more than 10 times lower in CD4+CD28^null^ T lymphocytes as compared to CD4+CD28+ T lymphocytes (Figure 4B). Only another gene, *DPP4* (dipeptidyl peptidase 4 also known as CD26 T cell activation costimulatory molecule) was detected with downregulation higher than five times in CD4+CD28^null^ T lymphocytes with respect to CD4+CD28+ T lymphocytes. The rest of the CD4+CD28^null^ downregulated genes showed expressions with differences lower than 5-fold. These last genes were *PLGC1/PLC* (phospholipase C gamma 1), *WASL/NWASP* (WASP-like actin nucleation promoting factor), *MYLK/MLCK* (myosin light chain kinase), *BCAR1/P130Cas* (BCAR1 scaffold protein belonging to the Cas family), *ACTN1/αActinin* (actinin alpha 1), *PIK3CA/PI3K* (phosphatidylinositol-4,5-bisphosphate 3-kinase catalytic subunit alpha), *VIM* (vimentin), *MYH10* (myosin heavy chain 10), and *EZR* (ezrin, a member of the ERM protein family) (Figure 4B).

All the tested genes in the array analysis were traced, and some of them were found to be involved in the regulation of different pathways related to leucocyte migration, such as focal adhesion, adherent junctions, leucocyte transendothelial migration, and regulation of the actin cytoskeleton. 

**Figure 5:**
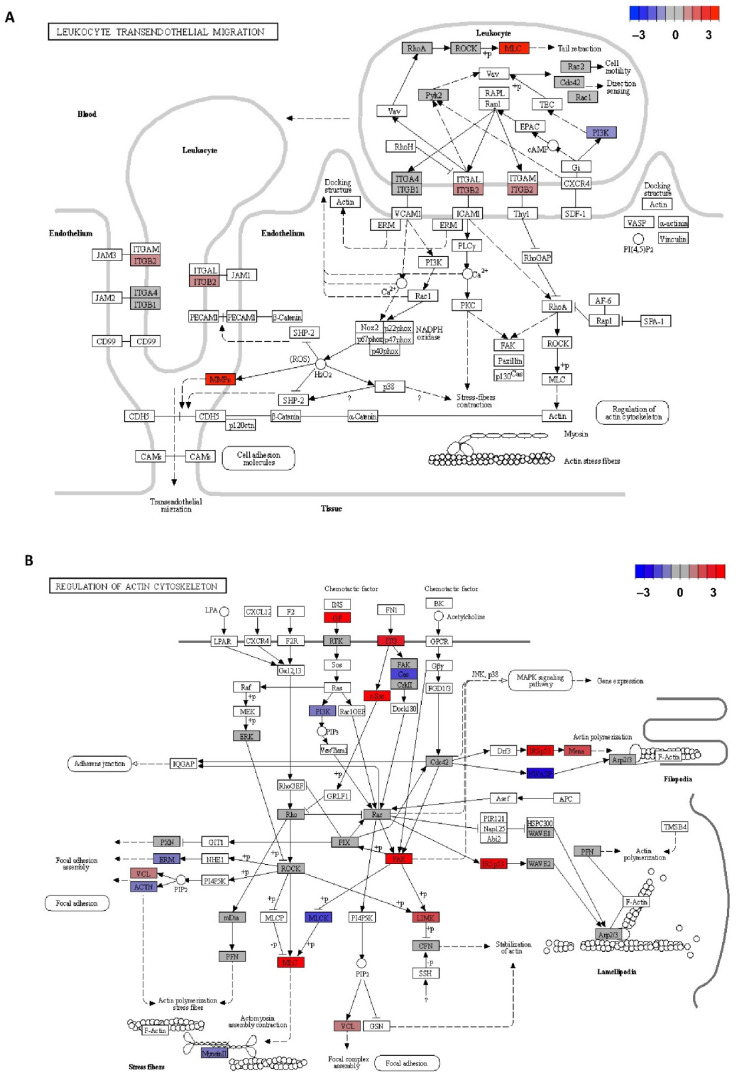
KEGG pathways representation with the differentially expressed genes in CD4+CD28^null^ T lymphocytes with respect to CD4+CD28+ T lymphocytes stand out with colour. (**A**) Leucocyte transendothelial migration pathway (hsa 04670 entry). (**B**) Regulation of actin cytoskeleton pathway (hsa 04810 entry). Log_2_ of the fold change extracted from migration array assays was calculated, and differentially expressed genes were represented in base of this value with a colour scale from dark red (upregulated genes in CD4+CD28^null^ T lymphocytes) to dark blue (downregulated genes in CD4+CD28^null^ T lymphocytes). Arrows mean activation. Dashed arrows mean indirect effect. Lines mean binding. Blunt end lines mean inhibition.

Figure 5A represents the leucocyte transendothelial migration pathway where three of the significantly upregulated genes in CD4+CD28^null^ T lymphocytes with respect to CD4+CD28+ T lymphocytes (*MYL9/MLC*, *ITGB2,* and *MMP*) were found to be part of the genes expressed at the leucocyte compartment in this pathway (genes represented in red). On the other hand, the *PI3K* gene was also located in this pathway, which is slightly downregulated in CD4+CD28^null^ T lymphocyte genes (represented in blue). 

Figure 5B, in the same way, represents another important pathway for leucocyte migration as is the regulation of the actin cytoskeleton. In this case 10 of the genes involved in the pathway were shown as upregulated in CD4+CD28^null^ T lymphocytes compared to CD4+CD28+ T lymphocytes (*GF*, *ITG*, *SRC*, *PAK*, *IRSp53*, *ENAH/MENA*, *LIMK*, *VCL,* and *MLC*). On the other hand, six genes downregulated in CD4+CD28^null^ T lymphocytes were located in this pathway (*BCAR1* (belonging to the Cas family), *PI3K*, *NWASP*, *MLCK*, *EZR* (belonging to ERM protein family), and *ACTN*).

**Figure 6:**
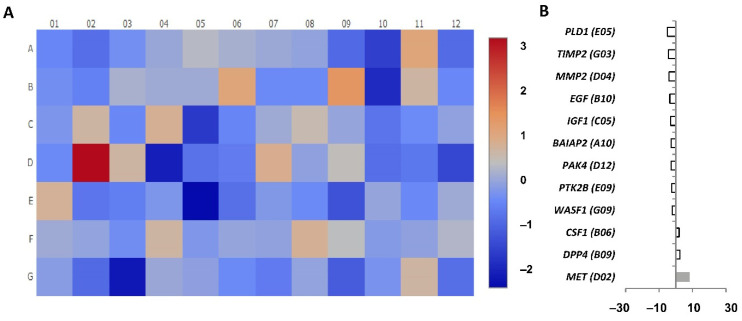
Cell motility array results comparing CD4+CD28^null^ IL-15 stimulated (test sample) with CD4+CD28^null^ basal condition (control sample) testing the expression of 84 different genes. (**A**) Heatmap performed by calculating the log_2_ of the fold change for each gene. The figure represents the level of expression of each gene with a colour scale from dark red (more expression in CD4+CD28^null^+IL-15) to dark blue (less expression in CD4+CD28^null^+IL-15). (**B**) Representation of the genes with significant expression differences. Grey bars represent fold regulation of genes with an expression difference higher than five but lower than 10. White bars represent fold regulation of genes with an expression difference higher than two. The wells corresponding to each gene are in brackets.

Gene expression was also compared between cells unstimulated and in response to IL-15. Firstly, we analysed array experiments carried out in CD4+CD28^null^ T lymphocytes at basal conditions compared with CD4+CD28^null^ T lymphocytes after IL-15 stimulation. In order to determine the possible changes in migration gene expression produced by IL-15 stimulation, as it was made for the previous analysis, fold regulation was calculated, taking in this case CD4+CD28^null^ T lymphocytes with IL-15 stimulation as the test sample and CD4+CD28^null^ T lymphocytes at basal conditions as the control sample (Figure 6).

In this case, just 12 of the genes analysed showed significant differences in the expression between the two conditions, and none of these genes showed a change of expression larger than 10-fold (Figure 6A). Only three of these genes showed significantly higher-level expression with IL-15 stimulation with respect to unstimulated cells (fold regulation > 2). Only one of these three genes, *MET* (MET proto-oncogene), which is a member of the receptor tyrosine kinase family, was overexpressed more than 5-fold. The other two upregulated genes were *DDP4* and *CSF1* (colony stimulating factor 1) and showed an expression change of less than 5-fold. Ultimately, nine genes were detected as significantly downregulated with IL-15 stimulation with respect to unstimulated CD4+CD28^null^ T lymphocytes (fold change < −2). Notice that all of these downregulated genes had a slight downregulation (fold change lower than 5-fold). These downregulated genes were *WASF1* (WASP family member 1), *PTK2B* (protein tyrosine kinase 2 beta), *PAK4* (p21 activated kinase 4), *BAIAP2/IRSp53*, *IGF1*, *EGF*, *MMP2* (matrix metallopeptidase 2), *TIMP2,* and *PLD1* (phospholipase D1) (Figure 6B).

These results reflect that there are almost no differences in expression levels of the studied genes related to migration pathways when basal conditions and IL-15 stimulation on CD4+CD28^null^ T lymphocytes were compared. The few differences found were quite slight with respect to those observed in the comparison between CD4+CD28^null^ and CD4+CD28+ T lymphocytes. Moreover, some of the genes showed opposite regulation than those found when CD4+CD28^null^ T lymphocytes and CD4+CD28+ T lymphocytes were compared (*TIMP2*, *EGF*, *IGF1*, *BAIAP2*, and *DPP4*), which makes it very unlikely that they are linked to the activating effects of IL-15 on migration that were previously described at the cellular level.

Lastly, when the effect of IL-15 on CD4+CD28+ T lymphocytes was assayed, very slight differences were found. The decrease was remarkable in *ACTN3* (fold change >10) and in *MMP2* (fold change > 5), the latter was also found when CD4+CD28^null^ T lymphocytes were treated with IL-15. Moreover, an increase in *DPP4* expression occurred in both cellular subsets in response to IL-15 (Figure S1).

As it is seen in these results, differences at gene expression level found between CD4+CD28 null and CD4+CD28+ T cells with IL-15 stimulation are quite consistent with those found between these T cell subsets without stimulation, so this fact supports the reliability of our gene expression results. 

### 3.4. Effect of IL-15 on Rho Family GTPases Activity in CD4+CD28^null^ and CD4+CD28+ T Lymphocytes

Rho-GTPases (RhoA, Rac 1, and Cdc42) are important migration-related proteins that control the dynamics of the cytoskeleton. We considered the activity of these proteins relevant to the study, since we found several genes related to the actin cytoskeleton pathway differently regulated between CD4+CD28^null^ and CD4+CD28+ T lymphocytes. In these experiments, protein extract pools were performed on 10 RA patients. After isolation, both cell subsets were starved for 1 h in serum-free medium and then stimulated 5 min with IL-15. After this stimulation, proteins were obtained and mixed in pools.

**Figure 7:**
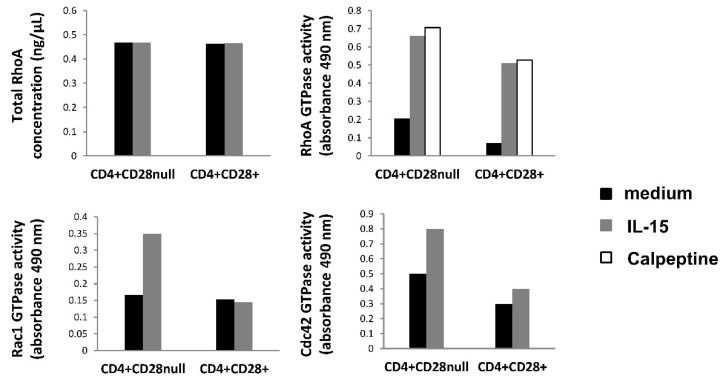
GLISAS analysis. CD4+CD28^null^ and CD4+CD28+ T cells were separated and, after 1 h of starvation, were incubated with and without IL-15 (50 ng/mL) stimulation. Protein extracts were obtained from each condition, and GLISAS experiments were made with them. Upper histograms show the results obtained for the RhoA protein; the left one represents the total protein RhoA concentration; the right one shows the GTPase activity of RhoA. Bottom histograms represent the GTPase activity of Rac1 (left) and Cdc42 (right). Basal conditions are represented in black; IL-15 stimulation conditions are in grey, and calpeptin positive control of RhoA activity in white.

RhoA basal levels were quantified in the two T lymphocyte subsets under both conditions, and we did not find differences in protein levels between them (Figure 7). Nevertheless, the basal activation of RhoA was slightly higher in CD4+CD28^null^ T lymphocytes than in CD4+CD28+ T lymphocytes, but in both subsets, IL-15 showed an important activator effect. Notice that the RhoA activity in each T lymphocyte subset is similar when cells were treated with IL-15 and were stimulated with calpeptin (activation control condition). On the contrary, Rac1 GTPase did not display basal activation differences between CD4+CD28^null^ T lymphocytes and CD4+CD28+ T lymphocytes, and IL-15 only exerted an activator effect on the first one (Figure 7). In the case of Cdc42, the protein showed a higher degree of activation in CD4+CD28^null^ T lymphocytes than in CD4+CD28+ T lymphocytes both at the basal level and in response to IL-15 (Figure 7).

In conclusion, these results reflect that activation of these proteins, RhoA, Rac1 and Cdc42 (phosphorylated forms), were clearly higher in CD4+CD28^null^ T lymphocytes from RA patients in response to IL-15, whereas only RhoA was activated in CD4+CD28+ T lymphocytes.

In these assays, the total RhoA protein concentration is quite similar between all the analysed conditions, and this result is consistent with what is observed at the gene expression level, so it supports the reliability of these results. 

## 4. Discussion

CD4+CD28^null^ T lymphocytes represent a cell subset with special peculiarities that make them different from the rest of the T helper cells. For instance, they have increased effector functions triggering a higher inflammatory capacity [3]. The amount of this cell subset has been expanded in ageing and pathogenic situations, such as chronic viral infections and autoimmune diseases, being widely described in RA patients [4]. In RA disease, it has been shown that CD4+CD28^null^ T lymphocytes and IL-15 levels are significantly increased in the blood and synovial fluid of these patients, suggesting an active role of both of them in the maintenance of inflammation in the target tissue, which could be correlated with the immunopathology and clinical activity of the disease [9,10,11,12]. Based on all this, we decided to study the migratory capacity of these CD4+CD28^null^ T lymphocytes and the effect that IL-15 could have in this process. 

We have observed that the levels of expression of the adhesion molecules CD49d and CD11a and of the chemokine receptors CX3CR1 and CCR5, were higher in CD4+CD28^null^ T lymphocytes than in CD4+CD28+ T lymphocytes. Furthermore, a greater enhancing effect of IL-15 on expression of these molecules was also predominantly found in this T cell subtype. These results agree with the previously published studies demonstrating a greater expression of CD11a, CD49d and CX3CR1 in CD4+CD28^null^ T lymphocytes than in CD4+CD28+ T lymphocytes [18]. Specifically, CX3CR1, the receptor of fractalkine/CX3CL1, was one of the molecules much more expressed in CD4+CD28^null^ T lymphocytes than in CD4+CD28+ T lymphocytes. It has been demonstrated that fractalkine/CX3CL1 plays an important role in the development of RA, suggesting a more aggressive role of this molecule in RA pathogenesis, as well as an association with atherosclerotic damage in these patients. CD4+CD28^null^ T lymphocytes may participate in both processes, being attracted by fractalkine to the inflammatory sites [19,20]. In contrast to the study of Broux and collaborators made on MS patients [18], we did not find an increase in CX3CR1 expression in response to IL-15 treatment, which is probably due to the different culture times used. However, CCR5, LFA-1 (CD11a/CD18), and VLA-4 (CD49d/CD29) were positively regulated in both studies. Most of the effects on the migratory ability of lymphocytes caused by IL-15 have been demonstrated in NK cells, in which this cytokine acts as a chemotactic factor that stimulates adhesion of NK cells to endothelial cells. Moreover, it is known that IL-15 also enhances the transendothelial migration of T lymphocytes by the activation of LFA-1 binding capacity to its ICAM-1 ligand [21]. Therefore, IL-15 is an important factor for NK cell recruitment to the target tissues, and this is possibly reproduced by CD4+CD28^null^ T lymphocytes [22]. It should be noted that both cell types share other functional properties and surface expression of several markers. These results also agree with those observed in the gene expression array experiments where the gene *ITGB2* coding CD18 protein, which can form part of the LFA integrin, was found overexpressed in CD4+ CD28^null^ T lymphocytes when compared with CD4+CD28+ T lymphocytes. 

Apart from it, other genes importantly related to migration have an upregulated expression in CD4+CD28^null^ T lymphocytes when compared with CD4+CD28+ T lymphocytes.

*MMP9* was the gene with the highest expression difference detected between CD4+CD28^null^ T lymphocytes and CD4+CD28+ T lymphocytes, being its expression almost 30-fold higher in CD4+CD28^null^ cells. Lymphocyte binding to the endothelial cell during the transmigration process is mediated by receptors, such as vascular cell adhesion molecule 1 (VCAM-1). As is shown in Figure 5A, this interaction induces NADPH oxidase activation and the production of reactive oxygen species (ROS) in a Rac-mediated manner, with subsequent activation of MMPs and loss of VE-cadherin-mediated adhesion [23]. It is described that MMP9 and MMP2 are the metalloproteinases that most contribute to protease activity associated with infiltrating leucocytes [24]. Specifically, MMP9 or gelatinase B is known to be produced by polymorphonuclear leucocytes, and it was the metalloproteinase activity first detected in rheumatoid synovial fluid [25]. Therefore, it is not a surprise that the gene coding this protein was overexpressed in CD4+CD28^null^ T lymphocytes which as was said above, is an expanded subset in autoimmune diseases, such as RA. 

The *SRC* proto-oncogene was detected in CD4+CD28^null^ T lymphocytes with an almost 20-fold increase as compared to that in CD4+CD28+ T lymphocytes. This proto-oncogene may play a role in the regulation of embryonic development and cell growth, and it also has been seen to be involved in the promotion of some tumours and their malignant progression [26]. From an immunological point of view, *SRC* has been seen to be involved in TLR2 phosphorylation, which triggers Ca^2+^ fluxes, and it has been postulated that through this signalling event the cell junctions could be modified to facilitate the migration of polymorphonuclear leucocytes across the epithelial barrier [27]. It is noteworthy that TLRs, although not typical of T lymphocytes, are expressed in certain T lymphocyte subpopulations, such as CD4+CD28^null^, where TLR4 and TLR2 have been found [28,29].

*PLAUR*, which is a urokinase-type plasminogen activator receptor, was another gene whose expression was upregulated more than 10-fold in CD4CD28^null^ T lymphocytes. Different studies indicate that Plaur protein plays a key role in promoting neutrophil adhesion and their transendothelial migration for recruitment into inflamed tissues [30,31].

Otherwise, Rho GTPases, RhoA, Rac, and Cdc42, are core proteins of the actin cytoskeleton regulation necessary for cell migration, as is seen in Figure 5B. In the classic model of cell migration, it is well-known that the GTPases Rac and Cdc42 are active at the leading edge of the cell, promoting bulge formation, whereas RhoA GTPase is only active in the cell body and at the rear in order to provide the actomyosin-mediated force needed for forward movement. Although, in our experiments, none of these GTPase genes were found as differentially expressed, some of the downstream targets of these proteins were detected as upregulated in CD4+CD28^null^ T lymphocytes as compared with CD4+CD28+ T lymphocytes. Moreover, in the GLISAS experiments, we detected enhanced activity of the three Rho GTPases after IL-15 stimulation in CD4+CD28^null^ T lymphocytes, while this treatment triggered the increased activity of Cdc42 in CD4+CD28+ T lymphocytes. Specifically, Rac proteins are necessary for promoting lamellipodia formation, which are cytoskeletal actin projections on the leading edge of the cell necessary for cell migration [32]. Some of the genes regulated by Rac were detected as upregulated in CD4+CD28^null^ T lymphocytes as compared with CD4+CD28+ T lymphocytes. This is the case with *IRSp53*, also known as *BAIAP2*, whose coded protein is an essential intermediate between Rac and WAVE. WAVE complex proteins act by stimulating rapid actin polymerization through the Arp2/3 complex (actin-related proteins 2 and 3) [33]. 

PAK and LIMK are also proteins regulated by Rac whose coding genes were found upregulated in CD4+CD28^null^ T lymphocytes. These two proteins exert their control over cofilin (CNF) whose function is to perform the actin turnover of the microfilaments of the cytoskeleton. The Rac/PAK signalling pathway also regulates myosin light chain (MLC/MYL9) phosphorylation, which increases actomyosin contractility at the cell periphery, although MLC activation is mainly triggered by the Rho/ROCK signalling pathway. Again, the *MLC* gene was found upregulated in CD4+CD28^null^ cells, in this case more than 5-fold. Thus, Rac/PAK and Rho/ROCK signalling triggers cofilin inactivation on the one hand and myosin II activation through the MLC pathway on the other hand, causing overall actin cytoskeleton reorganization that leads to stress fibre assembly and lamellipodium formation in the cell periphery. These alterations in cell morphology are performed in order to favour cell migration [34]. Besides this, in this study, significant differences were also found in the gene expression of some of the downstream targets of Cdc42 GTPase. The results suggest that, in CD4+CD28^null^ cells, Cdc42 exerts its function preferably by activating Arp2/3 through the IRSp53-Mena pathway better than by the alternative NWASP pathway, since the *IRSp53* and *MENA* genes were found to be upregulated, while *NWASP* was downregulated in this T lymphocyte subset. The IRSP53-MENA complex triggers actin filament assembly into filopodia, which are cytoplasmatic projections that extend beyond the leading edge of the lamellipodia in migrating cells [35].

Growth factors have been seen as frequent outside activators of this actin cytoskeleton regulatory signalling pathway necessary for cell migration [36] (Figure 5B). In the present study, it is seen that several growth factors genes are found upregulated in CD4+CD28^null^ T lymphocytes, as in the case of *FGF2*, *GFI1*, *HGF*, *EGF,* and *VEGFA*. These findings could suggest that migration of CD4+CD28^null^ cells could be induced by these different growth factors that might act in an autocrine manner. Specifically, HGF/Met induces the proliferation and migration of endothelial and tumour cells through RhoA, Rac1, and Cdc42 activation [37]. *HGF* expression has been found increased in CD4+CD28^null^ T lymphocytes, whereas *MET* was the gene found with the most increased expression in these cells in response to IL-15 stimulation. Recently, it has been found that a fraction of murine cytotoxic lymphocytes expresses c-Met and displays augmented cytolytic activity [38]. It is important to take into account that IL-15 enhances the cytotoxic properties of CD4+CD28^null^ T lymphocytes, and c-Met may be implicated in this regulation.

Taking all these results together, it seems clear that CD4+CD28^null^ lymphocytes are overexpressing essential genes for remodelling the actin cytoskeleton, specifically some of the downstream targets of RhoA, Rac, and Cdc42 GTPases. This fact can partially explain the higher migratory capacity of these cells, and as it is shown by these gene expression experiments, it is set from a transcriptional level.

Other interesting data extracted from this study are the downregulated genes observed in CD4+CD28^null^ lymphocytes with respect to CD4+CD28+ lymphocytes. Among these genes, the most downregulated gene is protein kinase C alpha (*PRKCA/PKCA*) with an expression difference of almost 20-fold. PKC family members phosphorylate a wide variety of protein targets. Among other functions, PKCA protein is involved in the regulation of the immune response of CD4+ T cells, and specifically, it has been implicated in T cell receptor/CD28-induced interleukin 2 gene expression [39]. This fact could explain why this gene is downregulated in cells without CD28 expression. PKCA has also been involved in migration signalling through a VCAM-1-dependent pathway [40,41]. Either way, the migration capacity of CD4+CD28^null^ cells seems to be PKCA-independent.

*DDP4*, also known as CD26, is another downregulated gene in CD4+CD28^null^ T lymphocytes, although IL-15 induced an increase in *DDP4* expression in both subsets of CD4+ cells. The protein coded by this gene is a T lymphocyte costimulator, with dipeptidyl peptidase enzymatic activity implicated in the development, maturation and migration of CD4+ T lymphocytes [42]. It is interesting that different previous reports have shown that CD26+ T lymphocytes exhibit strong migratory ability through endothelial cells, and these cells have been detected at high levels in the synovium and synovial fluids of RA patients, suggesting a role in inducing the inflammation and tissue destruction of these cells [43,44]. Delving into this, it has already been reported in progressive multiple sclerosis patients that this CD26 T lymphocyte costimulator is mainly expressed in CD28+ T lymphocytes, and its expression is much lower in CD4+CD28^null^ T lymphocytes [45]. Therefore, comparing our data with this, it seems that the CD26 costimulator is minimally expressed in CD4+CD28^null^ cells, but it is overexpressed in their counterpart, CD4+CD28+ T lymphocytes, in RA patients. 

As it was described in Section 3, our RT-PCR array experiments also reflected that there are almost no differences in the gene expression levels of the migration genes studied between basal conditions and IL-15 stimulation on CD4+CD28^null^ T lymphocytes. Something similar was observed with IL-15 stimulation of CD4+CD28+ T lymphocytes (Figure S1). This finding seems to suggest that the role that IL-15 has in lymphocyte motility, specifically in CD4+CD28^null^ T lymphocytes, which were observed with other experiments, could not be regulated at the transcription level. It is quite possible that the migration effects observed with IL-15 are then regulated through post-transcriptional and post-translational modifications. 

An important point to highlight from all these gene transcription results is the fact that none of the Rho GTPase genes showed any expression differences between the two different CD4+ T lymphocyte compartments. This fact fits with the RhoA basal levels measured in this work, which also reflected no differences between any of the conditions compared. Furthermore, our GLISAS experiments reflect that the activities of the proteins (RhoA, Rac1, and Cdc42) were higher in CD4+CD28^null^ T lymphocytes than in CD4+CD28+ T lymphocytes, and this basal activity increased after IL-15 stimulation, especially in CD4+CD28^null^ T lymphocytes. Hence, putting all these findings together, it may indicate that these proteins must be activated in these conditions by the post-translational mechanisms, which are already known for these RhoA GTPases [46].

Until now, only one work that studied the effect of IL-15 on the migration capacity of the CD4+CD28^null^ T lymphocytes has been published, and in that case, it was made in multiple sclerosis patients [18]. In this report, Broux and collaborators observed that IL-15 has more effect in CD4+CD28^null^ T lymphocytes than in CD4+CD28+ T lymphocytes, with regards to increasing their migration capacity. This migration capacity allows these cells to infiltrate the target tissue where they might trigger inflammatory damage. Our results agree quite well with this study, suggesting that this increase in the migration capacity probably depends on the T lymphocyte population itself more than on the particular disease. This fact makes it possible to extrapolate these results to any situation where the CD4+CD28^null^ T cell subset is expanded, as is the case with multiple sclerosis or RA.

## 5. Conclusions

In this work, it was demonstrated that CD4+CD28^null^ T lymphocytes, expanded in RA, showed more migration capacity than CD4+CD28+ T lymphocytes. We determined that the high migration capacity of CD4+CD28^null^ cells is being importantly controlled at gene expression level of several genes that are central in important signalling pathways related to cell migration. This migration capacity was seen to be increased by IL-15 stimulation, especially in CD4+CD28^null^ T lymphocytes. However, this change was not so apparent at the gene expression level. Finally, in the present work, we have determined a large list of genes and proteins that might be implicated in the migration of CD4+CD28^null^ T lymphocytes and could play a role in the pathogenesis of RA. It is quite probable that the enhanced migratory capacity of these cells allows them to reach the target tissue and perpetuate the inflammatory damage. Although the pathogenic role of CD4+CD28^null^ T lymphocytes in RA has not been yet firmly established and further research is necessary, these genes and proteins could be investigated as possible therapeutic targets for RA in future research.

## Data Availability

The data used in this work will be available in Gene Expression Omnibus (https://www.ncbi.nlm.nih.gov/geo/info/submission.html, accessed on 7 June 2021).

## Supplementary Materials

The following are available online at https://www.mdpi.com/article/10.3390/jpm11070594/s1, Figure S1: Supplementary Figure S1, Table S1: Supplementary Table S1.
