# Supplementary figures and images for: Acquisition of New Migratory Properties by Highly Differentiated CD4+CD28null T Lymphocytes in Rheumatoid Arthritis Disease
Source: J Pers Med. 2021 Jun 24;11(7):594. doi: 10.3390/jpm11070594 (PMC8306508; doi:10.3390/jpm11070594)

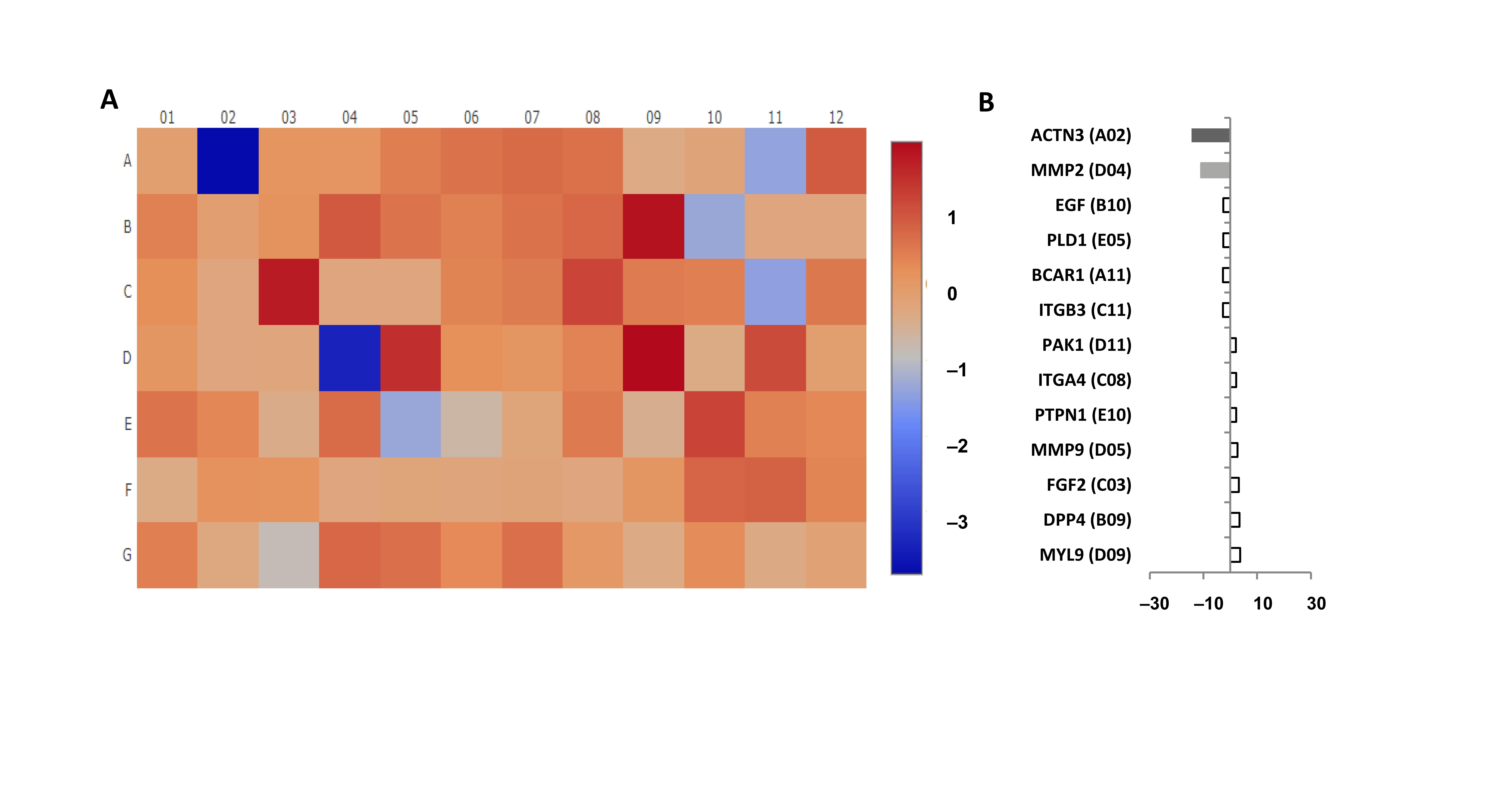

Supplement: Supplementary file 1 [file jpm-11-00594-s001.zip › Figuras PDF-8_01.jpg]
